# Investigating the prevalence of mental disorders and related risk factors in refugees and asylum seekers in Campania

**DOI:** 10.1192/j.eurpsy.2023.638

**Published:** 2023-07-19

**Authors:** L. Giuliani, D. Palumbo, G. M. Giordano, A. Perrottelli, P. Pezzella, E. Caporusso, P. Bucci, G. Corrivetti, G. Storti, F. Piras, R. Bracalenti, A. Mucci, S. Galderisi

**Affiliations:** ^1^Department of Psychiatry, University of Campania “Luigi Vanvitelli”, Naples; ^2^Department of Mental Health; ^3^UOC Area della Fragilità, ASL Salerno, Salerno; ^4^Department of Clinical and Behavioral Neurology, IRCCS Santa Lucia Foundation; ^5^Istituto Psicoanalitico per le Ricerche Sociali, Rome, Italy

## Abstract

**Introduction:**

In recent years the increasing presence of refugees and asylum seekers displaced from their country of origin, determined significant social, economic, humanitarian and public health implications in host nations. Advancing the knowledge on factors contributing to these implications, could foster the implementation of new public-health plans for these population. As a matter of fact, to date, the rates of mental disorders in these population are uncertain due to the high variability of methods used in the studies on topic, and of risk and protective factors analyzed. The most replicated finding is the high prevalence of Post-Traumatic Stress Disorder (PTSD) and depression in refugees and asylum seekers as compared to the population of host countries.

**Objectives:**

The aim of the present study was to investigate the needs for mental health prevention, care and rehabilitation of adult refugees and asylum seekers in Italy, performing a multidisciplinary evaluation of migrants who were guests in two refugees’ centers in Campania (Salerno and Avellino).

**Methods:**

The Mini-International Neuropsychiatric Interview (MINI) was assessed in 303 migrants, in order to evaluate the presence or not of a psychiatric diagnosis. Analysis of variance (ANOVA) was used to investigate differences between migrants with a mental disorder vs migrants without a mental disorder in terms of cognitive functions, depressive and anxiety symptoms, traumatic events and pre-migration risk factors. Person’s correlation was performed to investigate relationships between the Hopkins Symptom Checklist-25 (HSCL-t25) psychopathological index with all the other above-mentioned variables. Logistic regression was used to evaluate factors associated to the presence of a current mental disorder.

**Results:**

At least one mental disorder was found in 90 subjects (29.7% of the sample). Most prevalent diagnoses were major depressive disorder, lifetime panic disorder, PTSD, and generalized anxiety disorder. People with at least one psychiatric illness showed impaired global (F=6.62; p=.011) and social (F=8.22; p=.004) cognition, higher trauma levels (F=70.59; p<.0001) and more severe anxiety and depressive symptoms (F=61.84; p<.0001) compared to healthy migrants. Only trauma levels significantly correlated with HSCL-t25 psychopathological index. Trauma levels, global cognition, occupation, and migration status were associated to the presence of a current mental disorder.

**Conclusions:**

The results of the present study demonstrated that almost 1/3 of the guests of refugee centers in Campania have a mental disorder. The identification of risk factors associated to the onset of mental disorder and to severity of psychopathology in refugees and asylum seekers, may contribute to plan preventive and early psychiatric care in this population.

**Disclosure of Interest:**

None Declared

